# Integrated Management of Tomato Fusarium Wilt: Ultrastructure Insights into Zn Nanoparticles and Phytohormone Applications

**DOI:** 10.3390/cells14141055

**Published:** 2025-07-10

**Authors:** Yasmin M. Heikal, Amal M. Albahi, Amal A. Alyamani, Hala M. Abdelmigid, Samia A. Haroun, Hoda M. Soliman

**Affiliations:** 1 Botany Department, Faculty of Science, Mansoura University, Mansoura 35516, Egypt; amalmokhtar230@gmail.com (A.M.A.); samiaharoun@yahoo.com (S.A.H.); hudasoliman@mans.edu.eg (H.M.S.); 2 Department of Biotechnology, College of Science, Taif University, Taif 21944, Saudi Arabia; a.yamani@tu.edu.sa (A.A.A.); h.majed@tu.edu.sa (H.M.A.)

**Keywords:** wilt disease, *Fusarium oxysporum*, *Solanum lycopersicum*, control strategies, anatomical adaptations, ultrastructural changes

## Abstract

Fusarium wilt (FW), induced by *Fusarium oxysporum*, poses a significant threat to global tomato (*Solanum lycopersicum* L.) production, leading to substantial yield reduction. This study investigated the anatomical and ultrastructural responses of tomato leaves to FW infection and assessed the efficacy of salicylic acid (SA), humic acid (HA), and zinc oxide nanoparticles (ZnO-NPs) as control and inducer agents. FW infection resulted in notable structural alterations, including decreased leaf blade and mesophyll thickness and increased Adaxial epidermal cell wall thickness, thereby disrupting the leaf structure. Also, it caused severe chloroplast damage, such as membrane detachment and a reduced count of starch granules, which could impair photosynthetic efficiency. The different treatments exhibited significant effectiveness in reversing these adverse effects, leading to increased thickness of the leaf blade, mesophyll, palisade, and spongy tissues and enhanced structural integrity. Furthermore, ultrastructural improvements included activated mitochondria, compact chloroplasts with increased numbers, and proliferation of plastoglobuli, indicating adaptive metabolic changes. Principal component analysis (PCA-biplot) highlighted the significant parameters distinguishing treatment groups, providing insights into trait-based differentiation. This study concluded the potential of SA, HA, and ZnO-NPs as sustainable solutions for managing Fusarium wilt and enhancing tomato plant resilience, thereby contributing to improved agricultural practices and food security.

## 1. Introduction

Tomato (*Solanum lycopersicum* L., formerly *Lycopersicon esculentum* Mill.) is an annual herbaceous plant belonging to the Solanaceae family. Among tropical vegetable crops, it is one of the most extensively grown crops worldwide, surpassed only by potatoes [1]. Tomatoes have significant dietary and economic importance, serving as a high-value horticultural crop for local markets while improving nutritional standards [2]. Globally, tomatoes enjoy immense popularity because of their health benefits, including reduced risk of cancer, cardiovascular diseases, and osteoporosis [3]. Additionally, their low production costs make them profitable crops for farmers [4]. Despite their importance, tomatoes are highly susceptible to numerous fungal pathogens, including *Fusarium* spp. [5]. Major tomato diseases include early blight, anthracnose, Verticillium wilt, bacterial wilt, Fusarium wilt (FW), bacterial canker, and tomato spotted wilt, which negatively impact production worldwide [1]. The symptoms of tomato wilt disease are evident on the leaves and include vein clearing, yellowing, curling of margins, stunting, drying of leaves and stems, formation of adventitious roots, marginal necrosis, defoliation, and eventually, the death of the entire plant [6].

FW is a substantial threat to a wide range of crops, including commercial legumes, tomatoes, melons, maize, bananas, and oil palms. This disease was first identified in melon plants in the United States in 1930 and later in Canada in 1945 [7]. *F. oxysporum* is a soil-borne fungus that survives in the soil as dormant propagules called chlamydospores. The infection process begins when Fusarium spores germinate in the soil, enabling the fungal pathogen to penetrate the plant roots, typically through root tips or wounds caused by nematodes or other factors [8]. Once inside the plant, the fungus colonizes the vascular tissues, producing microconidia that obstruct the upward movement of water and nutrients in the xylem of the pseudostem. Fungal hyphae adhere to the root surface and secrete cell wall-degrading enzymes, facilitating intercellular growth through the root cortex and into water-conducting tissues, including the phloem and xylem [7]. As the pathogen advances into the stem, sieve cells attempt to inhibit the spread of conidia; however, spores germinate and the mycelium proliferates until the entire xylem system is obstructed [9]. This vascular blockage disrupts the water supply to leaves, resulting in wilting and ultimately plant death [5,7]. Following plant death, the fungus forms chlamydospores within the surrounding tissues, which are released back into the soil where they remain dormant until conditions favorable for germination arise [10].

Ultrastructural studies using light microscopy have been employed to compare the anatomical characteristics of the stems, flower petioles, and leaves of FOL-infected tomatoes to those of healthy plants. Infected tomato leaves exhibit significant anatomical modifications, including remarkable increases in leaf blade thickness, palisade and spongy tissue thickness, and midvein malformations. These changes resulted in an increased midvein length and width. Electron microscopy further revealed internal cellular alterations caused by pathogen infection, such as the disorganization of phloem tissue and cell thickening [11].

Further exploration is needed to understand the histopathological changes in tomato tissues infected with *Fusarium oxysporum* f. sp. *lycopersici* (FOL). This study hypothesizes that wilted tomato plants exhibit significant structural changes, particularly in FOL-infected leaves, which warrant detailed investigation. Although structural changes in tomato roots and stems caused by abiotic stressors have been documented [6], few studies have examined ultrastructural and cellular changes in tomato leaves infected explicitly by *F. oxysporum*. By enhancing the understanding of plant–pathogen interactions, these findings have the potential to inform strategies for developing resistant tomato cultivars, contributing significantly to sustainable agriculture.

According to L’Haridon et al. [12], Fusarium wilt is a difficult disease to control. Some of the initial management strategies include cultural control, physical control, and chemical control. Chemical fungicide application is often not effective enough to control Fusarium wilts and other soil-borne diseases, and recently, there has been global outcry and public concerns about their deleterious effects [13]. However, the sustainable use of fungicides in FOL management is difficult due to the development of resistant isolates and damaging effects on the natural environment, the agro-ecosystem, and human beings [13]. Biological control offers a better alternative to the use of chemicals. It is the use of natural antagonistic organisms to combat pests or suppress plant diseases [14]. Nguyen et al. [15] focused on four phenolic-rich plant extracts (*Eucalyptus camaldulensis*, *Chromolaena odorata*, *Bidens pilosa*, and *Azadirachta indica)* and their mixtures to control *F. oxysporum* in vitro and in tomatoes.

This stimulated the development of alternative methods as disease management strategies. Plant resistance can be developed through the use of synthetic chemicals such as functional salicylic acid (SA) analogues, such as benzothiadiazole-7-carbothioic acid (acibenzolar-S-methyl) or BTH. It has been demonstrated that BTH induces systemic resistance in tomato roots and controls crown and root rot caused by *F. oxysporum* f. sp. *radicis-lycopersici* [16]. In addition, humic acid (HA) is a natural organic polymer formed through microbial activity and enzymatic decomposition of coal, peat, soil, and the remains of plants and animals. It contains various functional groups, including phenolic, carboxylic, and hydroxylic compounds [17]. HA also plays a role in shaping microbial communities in the rhizosphere and can influence the composition of extracellular secretions [18]. Furthermore, the use of nanotechnology in plant disease management offers promising potential by enabling precise and targeted delivery of active compounds to specific sites, thereby enhancing protection against pathogens [19]. Among these tools, the foliar application of zinc oxide nanoparticles (ZnONPs) has shown superior effectiveness compared to traditional zinc salt formulations, largely due to their improved leaf penetration capabilities [20].

In this investigation, salicylic acid (SA), humic acid (HA), and zinc oxide nanoparticles (ZnO-NPs) were selected because of their known roles in stress alleviation and plant defense activation. SA is a signaling molecule that regulates systemic-acquired resistance and enhances plant antioxidant activity, whereas HA improves nutrient uptake, soil properties, and overall plant growth. ZnO-NPs, on the other hand, are emerging as promising nanomaterials for enhancing plant immunity and protecting against biotic and abiotic stresses due to their antimicrobial properties and influence on enzymatic activities. These treatments were applied individually to evaluate their effectiveness in suppressing *F. oxysporum* infection and improving the anatomical and ultrastructural features of *S. lycopersicum* leaves. By leveraging these diverse approaches, this study provides valuable insights into sustainable strategies for managing FW in tomato.

## 2. Materials and Methods

### 2.1. Plant Materials

Tomato (*Solanum lycopersicum* L.) (Hybrid K 186 cultivar) seedlings, aged thirty-five days, were obtained from the Agriculture Research Centre (ARC) in Giza, Egypt. These seedlings were selected based on their uniform size and shape. The chemicals utilized in the experiment were either supplied by Sigma Chemical Company (Saint Louis, MO, USA) or locally sourced as analytical-grade reagents.

### 2.2. Experimental Design

Isolation and identification were mentioned in Albahi [21]. To culture *Fusarium oxysporum* f. sp. *lycopersici* (Fol), the fungus is first grown on either Potato Dextrose Agar (PDA) or a sand-cornmeal medium (950 g sterilized sand and 50 g coarsely ground cornmeal). The inoculated medium is incubated at a temperature of 25–28 °C for a period of 7 to 10 days, allowing for robust mycelial growth and sporulation. Once sufficient growth is observed, sterile distilled water is added to the culture plates, and the surface is gently scraped to dislodge the conidia. The resulting spore suspension is then filtered through sterile cheesecloth or gauze to remove any remaining mycelial fragments. Finally, the inoculum was prepared by combining *F. oxysporum* cultures with a sterilized medium and subsequently added to pots at a ratio of 1 g inoculum per 100 g soil. The seedlings were divided into eight equal groups and planted on 4 April 2024 in identical pots, each containing equal amounts of garden soil with a clay-to-sand ratio of 2:1 (*v*/*v*). Super phosphate was applied at a rate of 0.6 g per pot, in accordance with the recommended dosage for tomato plants (357.14 kg/ha) as specified by the Ministry of Agriculture of Egypt. The eight experimental groups were as follows:

T0−: Negative control (Dist. water);

T0+: Positive control (*Fusarium oxysporum*);

T1: SA (0.5 mM) + *F. oxysporum;*

T2: SA (0.6 mM) + *F. oxysporum;*

T3: HA (100 mg/L) + *F. oxysporum;*

T4: HA (150 mg/L) + *F. oxysporum;*

T5: ZnO-NPs (250 mg/L) + *F. oxysporum;*

T6: ZnO-NPs (500 mg/L) + *F. oxysporum.*

Treatments were applied via foliar spraying at 40 days of age (5 days after transplantation on 4 April 2024) after the eight-leaf appearance. Data was collected 35 days after transplanting (70 days old) at the early fruiting stage on 10 May 2024. Each treatment and control group (negative and positive, respectively) included six replicates. Leaves were collected at 70 days of age for light and transmission electron microscopy (TEM) examination.

### 2.3. Cytological and Ultrastructural Responses

#### 2.3.1. Light Microscopy

The specimen’s preparation was performed according to the modified protocol of Karnovsky [22]. Leaf blade tissue sections (1–2 mm^2^) from untreated and treated plants were preserved overnight in 0.1 M phosphate-buffered saline (PBS, pH 7.4) with 2.5% glutaraldehyde and 2% paraformaldehyde. Samples were washed with 0.1 M sodium phosphate buffer and 0.1 M sucrose for 15 min, post-fixed in 1% sodium phosphate-buffered osmium tetroxide (pH 7.4), dehydrated in ethanol, and embedded in Epon resin overnight at 4 °C. The resin was polymerized at 70 °C for three days. Semi-thin sections (0.1–0.5 μm) were prepared using a Leica EM UC7 ultramicrotome. Sections (~0.5 μm) were stained with 1% Toluidine Blue O (in 1% sodium borate) for 5 min, observed with an Olympus CX31RTSF microscope, and photographed using a ToupCam X Cam Full HD camera (ToupTck, Hangzhou, China). Two representative images from ten images per species were digitized.

#### 2.3.2. Measurements of Anatomical Parameters

The cross-sectional areas (midrib and interveinal lamina) of analyzed leaf parameters included total leaf thickness, epidermal thickness of abaxial and adaxial surfaces, mesophyll thickness, palisade thickness, vascular bundle area, phloem thickness, xylem element length, number of xylem conducting elements, and height and width of the vascular bundle and gland size. Measurements of leaf thickness, epidermis thickness on abaxial and adaxial margins, and mesophyll were conducted using the straight tool of Image J (https://imagej.nih.gov/ij/, accessed on 10 September 2024) on multiple consecutive sections (n = 25 aggregates/leaf), while vascular bundle areas were measured using the freehand selections tool of Image J.

#### 2.3.3. Transmission Electron Microscopy

Ultrathin sections, ranging from 50 to 100 nm, were meticulously prepared from the same Epon resin blocks utilized for semithin sections, using an ultramicrotome. The sections were stained with 2% uranyl acetate for 10 min and 1% lead citrate for 5 min, followed by a drying period of approximately 15 min according to Reynolds [23]. Ultrastructural analysis was conducted using a JEOL-JEM-2100 transmission electron microscope operating at 160 kV located at the EM Unit, Mansoura University, Egypt. TEM observations concentrated on the cell wall, chloroplasts, mitochondria, and other cellular ultrastructural components. Quantitative measurements included the chloroplast area, number of chloroplasts and mitochondria per cell, number of starch grains and plastoglobuli per chloroplast, and thickness of the cell wall between adjacent cells. The chloroplast area and starch granule area were determined by measuring the long axis and width of the chloroplasts and correlating their shapes to known geometric figures. All measurements were performed on the micrographs (5–10) obtained via TEM.

### 2.4. Statistical Analysis

Data pertaining to the semithin and ultrastructural parameters were collected using a random complete block design with three replications. Tests for normality and homogeneity of variance were conducted, followed by an analysis of variance (ANOVA) using SPSS (version 22.0, IBM Corp., Armonk, NY, USA) to examine variations among factors, with treatments serving as independent variables. Results are presented as mean ± standard error, with statistical significance set at *p* ≤ 0.05. For graphical representation, a cell plot was employed to integrate all studied parameters using JMP ver.16 (SAS Institute Inc., Cary, NC, USA, 2020–2021). Correlation coefficient matrices were computed to produce scatter plots and heat maps of semithin and ultrastructural parameters across treatments. Principal Component Analysis-biplot (PCA-biplot) was created using JMP ver.16.

## 3. Results

### 3.1. Semi-Thin Measurements

Light microscopic examination of semi-thin *S. lycopersicum* leaf sections revealed variations in mesophyll organization and leaf component thickness across treatments. The leaves had a dorsiventral structure with a uniseriate, thinly cutinized epidermis (Cu). The upper (adaxial) epidermis (Ad. ep) and lower (abaxial) epidermis (Ab. ep) contained stomatal openings (St) with two guard cells forming a stomatal chamber. The mesophyll layer comprises elongated palisade parenchymal cells (PP) beneath the adaxial epidermis and irregular spongy parenchymal cells (SP) below. Intercellular spaces (IS) were prominent in the spongy tissue. Vascular bundles (VBs) are distributed as secondary veins throughout spongy tissue or within the innermost palisade layer. These bundles consist of a phloem (Ph) and are composed of sieve elements and companion cells in parenchymal tissue and xylem (Xy), comprising short rows of thin tracheary components (2–4 elements).

**Figure 1:**
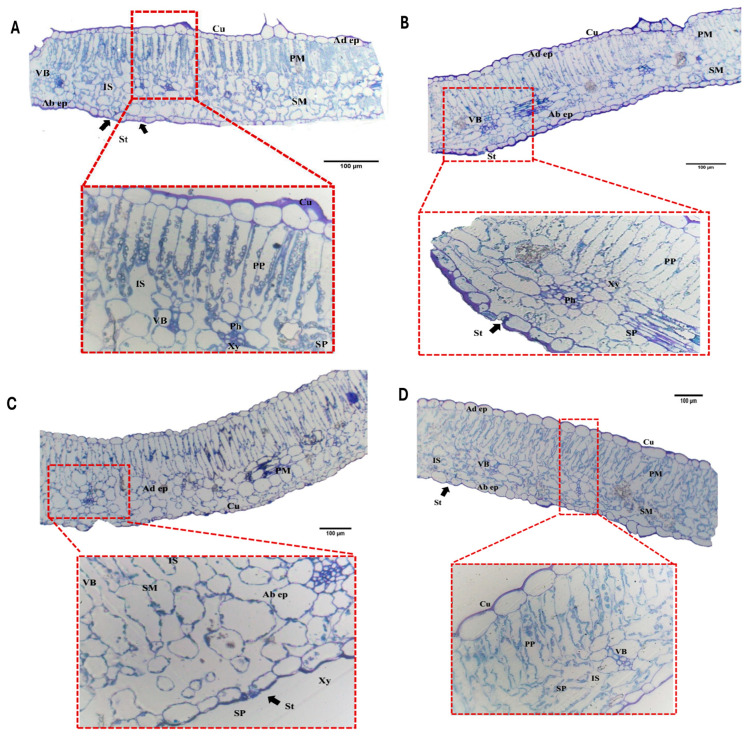
Photomicrographs of transverse sections of cultivated *S. lycopersicum* leaves showing leaf blade at 100× and its magnified part of photosynthetic palisade and spongy parenchymal cells and cuticle thickness at 400× under (**A**) absolute control conditions (T0−); (**B**) positive control conditions (T0+); (**C**) treated with 0.5 g/L salicylic acid (T1); (**D**) treated with 0.6 g/L salicylic acid (T2). Abbreviations: Black arrows showing the stomata opening; Cu, cuticle; Ad ep., adaxial epidermis; Ab ep., abaxial epidermis; PM, palisade mesophyll; SM, spongy mesophyll; VB, vascular bundle; Xy, xylem; Ph, phloem. PP, palisade parenchyma; SP, spongy parenchyma; IS, intercellular space; St, stomata; bar: 100 µm.

In the absolute control group (T0−), non-inoculated *S. lycopersicum* leaves showed well-organized anatomical and histological characteristics. The leaf blade thickness was 239.02 µm, with a cuticle thickness of 4.62 µm. The adaxial and abaxial epidermis thicknesses were 13.63 µm and 15.37 µm, respectively, while the mesophyll tissue thickness was 195.31 µm. The palisade and spongy tissue thicknesses were 82.18 µm and 100.46 µm, respectively. The palisade parenchymal cell area was 1049.79 µm^2^, the spongy parenchymal cell area was 711.29 µm^2^, and the vascular bundle area was 256.94 µm^2^ (Figure 1 and Table 1). After *F. oxysporum* infection (T0+), the leaf tissue expansion changed. The leaf blade thickness decreased to 212.71 µm, and the mesophyll thickness reduced to 179.81 µm. The reduction in spongy tissue thickness (76.51 µm) and spongy parenchymal cell area (422.08 µm^2^) contributed to leaf blade thinning. Increases were noted in cuticle thickness slightly (4.94 µm), adaxial epidermis thickness (15.86 µm), and abaxial epidermis thickness (16.31 µm). The palisade thickness increased to 96.16 µm, the palisade parenchymal cell area expanded to 1479.93 µm^2^, and the vascular bundle area increased to 351.18 µm^2^, indicating structural changes in response to infection.

Treatment with 0.5 g/L salicylic acid (T_1_) resulted in significant anatomical improvements in *S. lycopersicum* leaves affected by *F. oxysporum*. Histological analysis revealed that T_1_ increased the mesophyll parenchymal cell size, leading to thicker leaves (Figure 1) compared to the control group (T0−). Leaf blade thickness increased to 290.35 µm, mesophyll thickness to 258.40 µm, and palisade and spongy layer thicknesses to 144.39 µm and 115.49 µm, respectively. T_1_-treated leaves had the thinnest cuticle (2.40 µm) and reduced abaxial epidermis thickness (11.21 µm), while adaxial epidermis thickness increased to 19.59 µm. Palisade parenchymal cell area reached 2102.60 µm^2^, with spongy parenchymal cells (1203.83 µm^2^) and vascular bundles (768.79 µm^2^) also showing significant increases. Histological examination of leaves treated with 0.6 g/L salicylic acid (T_2_) showed reductions in leaf blade thickness (196.37 µm) and mesophyll thickness (169.98 µm) compared to the control and T_1_ leaves. Cuticle thickness decreased (2.58 µm), while adaxial and abaxial epidermal cell thicknesses increased to 24.32 µm and 19.97 µm, respectively. There was a reduction in palisade thickness (92.83 µm) and parenchymal cell area (967.65 µm^2^), along with spongy tissue thickness (77.47 µm) and parenchymal cell area (697.17 µm^2^). The vascular bundle area declined to 335.47 µm^2^ (Figure 1).

**Figure 2:**
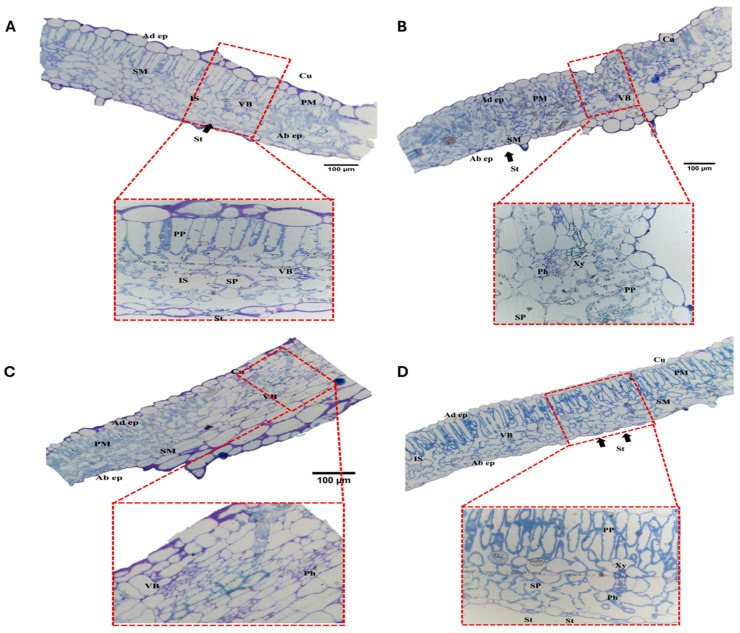
Photomicrographs of transverse sections of cultivated *S. lycopersicum* leaves showing leaf blade at 100× and its magnified part of photosynthetic palisade and spongy parenchymal cells and cuticle thickness at 400× under (**A**) treated with 100 mg/L of humic acid (T3); (**B**) treated with 150 mg/L of humic acid (T4); (**C**) treated with 250 mg/L of ZnONPs (T5); (**D**) treated with 500 mg/L of ZnO NPs (T6). Abbreviations: Black arrows showing the stomata opening; Cu, cuticle; Ad ep., adaxial epidermis; Ab ep., abaxial epidermis; PM, palisade mesophyll; SM, spongy mesophyll; VB, vascular bundle; Xy, xylem; Ph, phloem. PP, palisade parenchyma; SP, spongy parenchyma; IS, intercellular space; St, stomata; bar: 100 µm.

Treatment with 100 mg/L humic acid (T_3_) reduced leaf blade (188.93 µm), mesophyll (148.84 µm), palisade (65.35 µm), and spongy tissue thickness (73.28 µm). Abaxial epidermis thickness decreased (12.99 µm). Cuticle thickness (5.06 µm) reached a maximum value, and adaxial epidermis thickness increased to 23.48 µm compared to control leaves. Palisade parenchymal cell area (872.40 µm^2^) and vascular bundle area (164.28 µm^2^) reduced, while spongy parenchymal cell area increased to 815.76 µm^2^ (Figure 2).

Leaves treated with 150 mg/L humic acid (T_4_) showed increases in leaf blade (271.32 µm), mesophyll (227.42 µm), palisade (84.56 µm), and spongy tissue thickness (138.71 µm) compared to control and T_3_ leaves. Abaxial epidermis thickness increased to 26.59 µm, while cuticle (2.09 µm) and adaxial epidermis thickness (20.53 µm) decreased. Vascular bundle area reached the maximum of 1913.68 µm^2^, the palisade parenchymal area increased to 1068.66 µm^2^, and the spongy parenchymal cell area decreased to 677.14 µm^2^ (Figure 2).

Treatment with 250 mg/L ZnO-NPs (T_5_) resulted in increases in leaf blade (276.58 µm), mesophyll (220.48 µm), cuticle (4.78 µm), adaxial epidermis (23.37 µm), and abaxial epidermis thickness (26.56 µm). Spongy tissue thickness (160.85 µm) and parenchymal cell area (1614.09 µm^2^) increased due to vascular bundles measuring 1192.74 µm^2^. Palisade tissue thickness (54.02 µm) and parenchymal area (427.98 µm^2^) were reduced compared to control leaves (Figure 2). Leaves treated with 500 mg/L ZnO-NPs (T_6_) showed shrunken mesophyll cells, collapsed palisade cells, and angular-shaped spongy cells (Figure 2). This resulted in a thinner leaf blade (179.42 µm) and reductions in mesophyll (154.38 µm), palisade (74.62 µm), spongy tissue (72.97 µm), cuticle (0.78 µm), and abaxial epidermis thickness (8.90 µm) compared to control and T_5_ leaves. The palisade parenchymal cell area (602.64 µm^2^), spongy parenchymal cell area (428.85 µm^2^), and vascular bundle area (194.97 µm^2^) were decreased. Adaxial epidermis thickness increased to 18.40 µm (Figure 2).

### 3.2. Ultra-Structural Micrographs

**Figure 3:**
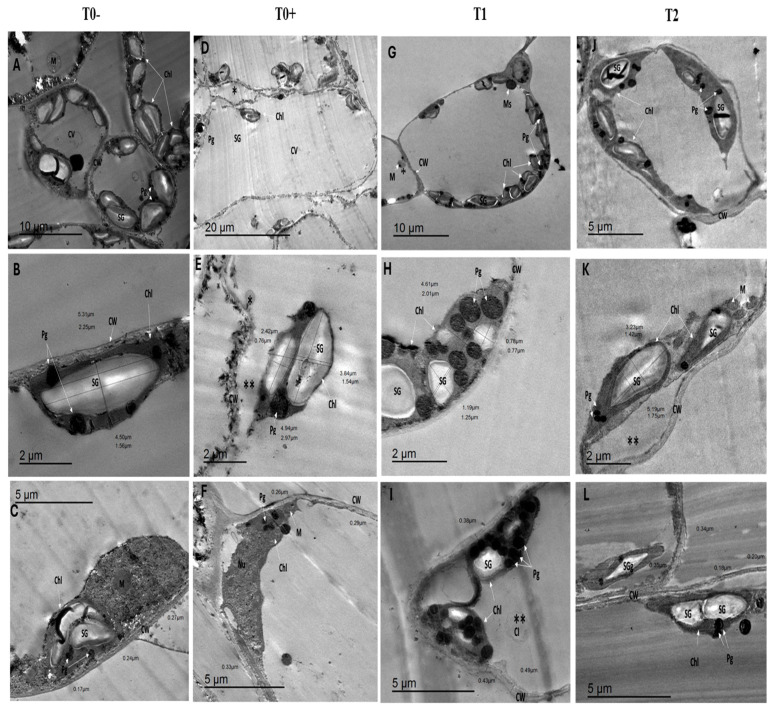
TEM micrographs of leaf ultrastructure of untreated *S. lycopersicum* plants under T0−, T0+, T1, and T2 conditions. (**A**) Mitochondria were intact, with complete membrane structure and clear mitochondria; (**B**) The elliptical chloroplast and the measured area of both chloroplast and starch granules; (**C**) Cell wall thickness and intact circular mitochondria; (**D**) The alerted shape of chloroplast and (*) the system of the membranes was obliterated; (**E**) Plasmolysis of cell membrane, (*) formation of cytoplasmic inclusion and (**) separation of chloroplast membrane from cell membrane; (**F**) Chloroplast without starch granules and alerted nucleus; (**G**) The size of chloroplast decreased and (*) mitochondria were cavitated completely; (**H**) The large-sized plastoglobuli and decreased starch granules of chloroplast; (**I**) The chloroplasts with numerous plastoglobuli, (**) cytoplasmic inclusions; (**J**) Chloroplast appeared elongated and detached from the plasma membrane; (**K**) Mitochondrial activity; (**) separation of chloroplast membrane from the cell membrane and (**L**) The elongated shape of chloroplasts with large-sized plastoglobuli and starch granules. Treatments: T0−: Negative control (dist. water); T0+: Positive control (*F. oxysporum*); T1: SA (0.5 mM) + *F. oxysporum* and T2: SA (0.6 mM) + *F. oxysporum*. Abbreviations: CW—cell wall, CV—central vacuole, Chl—chloroplast, M—mitochondria, Pg—plastoglobulus, SG—starch granules, CI—cytoplasmic inclusions, Ms—microsomes, Nu—nucleus. bars = 10, 2, and 5 μm.

**Figure 4:**
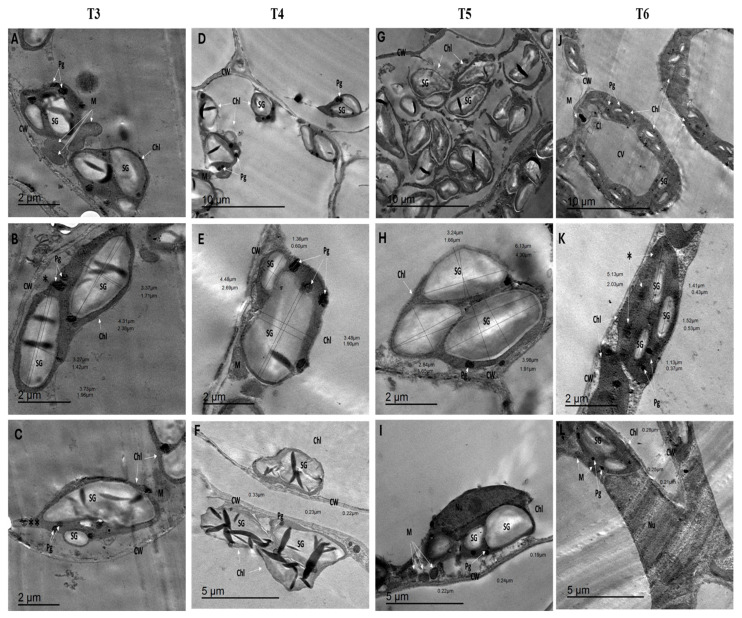
TEM micrographs of leaf ultrastructure of untreated *S. lycopersicum* plants under T3, T4, T5, and T6 conditions. (**A**) Different shapes of mitochondria (elongated and dumbbell); (**B**) large-folded starch granules, (*) detached chloroplast from the cell membrane; (**C**) detached chloroplasts with small and large-folded starch granules, (**) elliptical chloroplast with tail or projection. (**D**) Detached chloroplasts from the cell membrane with folded starch granules and with some projections; (**E**) swollen chloroplasts with different sizes, small and large folded starch granules; (**F**) irregularity of cell wall thickness and the large, folded starch granules. (**G**) Disintegration and rupture of chloroplast membrane and the folding of large-sized starch granules; (**H**) large size of chloroplast and starch granules; (**I**) normal nucleus and circular mitochondria with regular cell wall thickness. (**J**) The decreasing size of chloroplasts and starch granules and appearance of some cytoplasmic inclusions; (**K**) small size of chloroplast and starch granules, (*) irregular, disturbed grana; (**L**) normal nucleus and circular mitochondria with regular cell wall thickness. Treatments: T3: HA (100 mg/L) + *F. oxysporum*; T4: HA (150 mg/L) + *F. oxysporum*; T5: ZnO-NPs (250 mg/L) + *F. oxysporum* and T6: ZnO-NPs (500 mg/L) + *F. oxysporum*. Abbreviations: CW—cell wall, M—mitochondria, Ms—microsomes, Chl—chloroplast, Pg—plastoglobulus, SG—starch granules, CV—central vacuole, CI—cytoplasmic inclusions. Bars = 2, 5, and 10 μm.

Electron micrographs of *S. lycopersicum* leaves inoculated with *F. oxysporum* and subjected to various treatments are illustrated in Table 2 and Figure 3 and Figure 4. In the negative control group (T0−), leaves that were not inoculated exhibited typical microstructural characteristics, serving as a reference point for assessing the effects of different treatments (Figure 3A). Regarding the morphology of chloroplasts (Chl), the mesophyll cells in T0− plants contained ellipsoid-shaped chloroplasts with dimensions of 5.31 ± 0.27 µm in length, 2.25 µm in width, and an area of 9.89 µm^2^. Additionally, there were 9.25 starch granules (SG) per cell profile, with each granule measuring 4.50 µm in length, 1.56 µm in width, and covering an area of 5.35 µm^2^ (Figure 3B). The number of plastoglobuli (Pg) was 19.38 per cell profile, indicative of the typical metabolic condition of chloroplasts (Table 2). Other organelles and cellular structures in the T0 leaves displayed typical features. Approximately 1.50 small, normally sized mitochondria (M) were observed in each cell profile. The central vacuole (CV) appeared normal, and the cellular membrane was stretched and intact. The cell wall (CW) maintained its normal structure, with a thickness of 0.23 µm (Figure 3C). This served as a baseline for evaluating the microstructural changes induced by *F. oxysporum* infection and subsequent treatments.

The ultra-morphological characteristics of the positive control T0+ (treated with *F. oxysporum*) exhibited significant alterations compared to the T0− leaves. Notable changes were observed in the chloroplast regions of the leaves of positive control. Specifically, there was a separation of the chloroplast membrane from the cell wall, along with the disappearance of grana and starch grains in most chloroplasts, as illustrated in Figure 3D. Chloroplasts decreased in size, adopting a rounded shape with dimensions of 4.08 µm in length, 1.15 µm in width, and an area of 7.13 µm^2^. Additionally, the number of plastoglobuli increased to 21.33 per cell profile (Table 2), whereas the number of starch granules decreased to 3.33 per cell profile, with dimensions of 3.13 µm in length, 1.15 µm in width, and an area of 3.15 µm^2^, as depicted in Figure 3E. Furthermore, the cell membrane exhibited plasmolysis, and there was a disturbance in the cell wall, with its thickness increasing by 0.29 µm compared to T0 leaves. Moreover, the nucleus was separated and altered, and both the number and size of the mitochondria increased (Figure 3F).

The effects of salicylic acid treatment were observed in T_1_ and T_2_ leaves. The ultra-morphology of T_1_ leaves treated with salicylic acid (0.5 g) exhibited numerous cytoplasmic inclusions and a normal nucleus with a distinct nucleolus. The mitochondria appeared activated, characterized by markedly circular mitochondria, with a recorded number of 1.20 per cell profile (Figure 3G). The size and shape of chloroplasts were reduced, measuring 4.61 µm in length, 2.01 µm in width, and 7.35 µm^2^ in area. The chloroplast number was counted as 4.20 per cell profile. The number of starch grains decreased to 6.30 per cell profile, with dimensions of 0.99 µm in length, 1.01 µm in width, and an area of 0.75 µm^2^ (Figure 3H). In contrast, there was a noticeable increase in the size and number of plastoglobuli, recorded as 34.64 per cell profile (Table 2). Figure 3I shows that the cell wall thickness increased by 0.43 µm compared to the control leaves.

The ultrastructural morphology of T_2_ leaves treated with 0.6 g/L salicylic acid exhibited distinct variations compared to T_1_ and control leaves. Figure 3J illustrates an enhancement in mitochondrial activity, evidenced by an increase in both the number and size of mitochondria, with an average of 3.50 mitochondria per cell profile. The chloroplast (Chl.) count decreased to 3.60 per cell relative to T_1_. The chlorophyll appeared elongated and, in some instances, detached from the plasma membrane. The chloroplasts increased in size, measuring 5.69 µm in length, 2.41 µm in width, and 10.18 µm^2^ in area, compared to T_1_. The number of starch grains (SG) decreased to 4.80 per cell profile, although their area increased and exhibited folding, measuring 4.79 µm^2^ with dimensions of 3.44 µm in length and 1.87 µm in width, relative to T_1_ (Figure 3K). Additionally, the size and number of plastoglobuli (Pgs) decreased to 18.10 per cell in comparison with T_1_, as scored in Table 2. The cell wall (CW) was thicker than that of control leaves but thinner compared to T_1_, measuring 0.27 µm (Figure 3L).

The impact of humic acid treatments was evident in the ultrastructural alterations observed in the T_3_ and T_4_ leaves of *S. lycopersicum*. In T_3_ leaves (100 mg/L humic acid), mitochondria displayed diverse morphologies, with their number increasing to 3.00 per cell profile compared to control leaves (Figure 4A). Chloroplasts (Chl) appear swollen and round, occasionally exhibiting projections or tails. Some were detached from the plasma membrane, with their count at 3.64 per cell profile, smaller than control leaves, measuring 5.43 µm in length, 2.22 µm in width, and covering 8.65 µm^2^ (Figure 4B). Starch granules (SGs) were folded, with a 6.10 per cell profile, measuring 3.55 µm in length, 1.38 µm in width, and 3.53 µm^2^ area. Plastoglobuli (Pgs) decreased to 11.27 per cell. Cell wall (CW) thickness increased to 0.29 µm (Figure 4C).

In T_4_ leaves (150 mg/L humic acid), mitochondria were predominantly circular or dumbbell-shaped, increasing to 4.14 per cell (Figure 4D). Chloroplasts reached 7.64 per cell profile, measuring 5.23 µm in length, 2.72 µm in width, and 11.99 µm^2^ area. SGs doubled to 12.00 per cell profile, with large SGs measuring 7.31 µm^2^, 4.47 µm in length, and 1.93 µm in width, while small SGs measured 0.85 µm^2^, 1.36 µm in length, and 0.60 µm in width (Figure 4E). The Pgs count increased to 25.55 per cell. Cell wall thickness showed irregularities, measuring 0.27 µm (Figure 4F). This analysis highlights the significant changes induced by different humic acid concentrations, with notable improvements in the chloroplast and mitochondrial structure in T_4_ leaves.

The ultrastructural effects of ZnO nanoparticles (NPs) were examined in *S. lycopersicum* leaves subjected to treatments T5 and T6, focusing on changes in organelle morphology and cell wall structure. In T_5_ leaves (250 mg/L ZnO NPs), the mitochondria were circular, averaging 2.50 per cell profile. Chloroplasts increased to 4.13 per cell profile, with an area of 17.96 µm^2^, length of 6.13 µm, and width of 4.30 µm (Figure 4G). Chloroplasts contained more starch granules (SGs), totaling 8.00 per cell profile, with an area of 4.25 µm^2^, length of 3.61 µm, and width of 1.79 µm (Figure 4H). Despite the increased SGs, the chloroplast membranes showed disintegration and rupture. Plastoglobuli (Pg) count was estimated at 12.86 per cell profile. Cell wall (CW) thickness measured 0.22 µm (Figure 4I).

In T_6_ leaves (500 mg/L ZnO NPs), mitochondrial membrane rupture occurred, with the mitochondrial count decreasing to 2.20 per cell profile (Figure 4J). Chloroplasts reverted to an ellipsoid shape, decreasing to 4.00 per cell profile. The chloroplast area was reduced to 7.85 µm^2^, with a length of 5.13 µm and a width of 2.03 µm. SGs were smaller, with an area of 0.46 µm^2^, a length of 1.35 µm, and a width of 0.44 µm, and their number reduced to 6.50 per cell profile (Figure 4K). The Pg count increased to 23.00 per cell. Irregularly disturbed grana were present and separated from the chloroplast membranes. Normal nuclei and cytoplasmic inclusions were also observed. CW thickness measured 0.25 µm (Figure 4L). These findings demonstrate the differential effects of ZnO NP concentrations, with higher doses (T_6_) damaging the mitochondria and chloroplasts while increasing the plastoglobuli count.

### 3.3. Combined Inter-Correlation Among Semithin and Ultrastructure Traits of 70-Day-Old S. lycopersicum Plants Under the Different Eight Treatments

**Figure 5:**
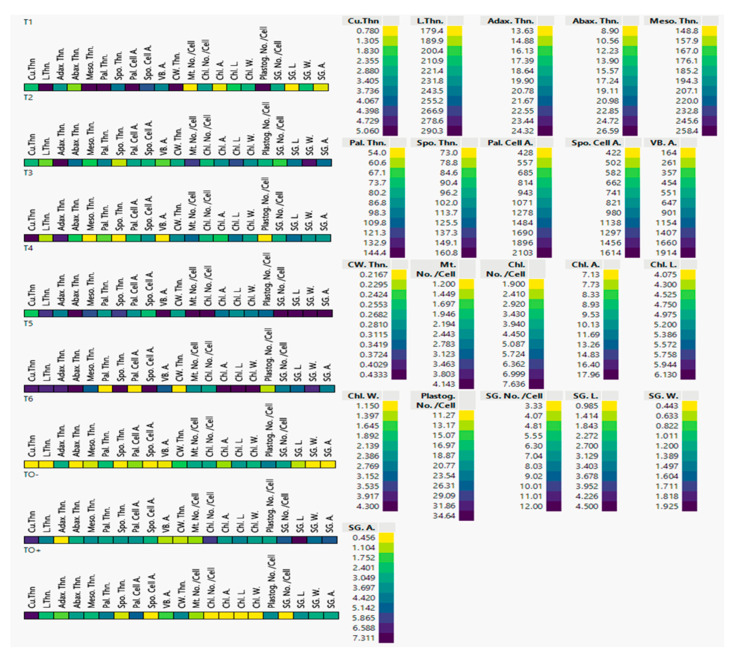
Cell plot of 21 semithin and ultrastructure traits of 70-day-old *S. lycopersicum* plants under the eight different treatments. Data represents means of three replicates; low levels are colored in bright yellow with greenish gradients and dark violet for high levels (see the legend on right side). Cu.Thn = cuticle thickness; L.Thn. = leaf thickness; Adax. Thn. = adaxial epidermis thickness; Abax. Thn. = abaxial epidermis thickness; Meso. Thn. = mesophyll thickness; Pal. Thn. = palisade thickness; Spo. Thn. = spongy thickness; Pal. Cell A. = palisade cell area; Spo. Cell A. = spongy cell area; VB. A. = vascular bundle area; CW. Thn. = cell wall thickness (µm), Mt. No./Cell = mitochondria number/cell profile; Chl. No./Cell = chloroplast number/cell profile; Chl. A. = chloroplast area (µm2); Chl. L. = chloroplast length (µm); Chl. W. = chloroplast width (µm); Plastog. No./Cell = plastoglobuli number/cell profile; SG. No./Cell = starch grains number/cell profile; SG. L. = starch grains length (µm); SG. W. = starch grains width (µm) and SG. A. = starch grains area (µm^2^).

Cell plot results of semithin and ultrastructure traits showed tremendous variations among 70-day-old *S. lycopersicum* plants under different treatments (Figure 5). In this study, 21 quantitative *S. lycopersicum* traits were tested; T0+ (Inoculated control), marked in dark violet, showed the lowest levels of Spo. Thn., Spo. Cell A., Chl. No./Cell, Chl. A., Chl. L., and SG. No./Cell. In contrast, T0+ showed the highest values of Cu. Thn., which was marked with bright yellow, and the values of the rest of the parameters were moderate greenish colors between yellow and violet. T0− (negative control) showed the contrary value of the tested parameters of T0+. T0− values ranged from moderate to high in all parameters except Adax. Thn., VB. A., CW. Thn., and Mt. No. Cells. T4 < T5 < T2, which showed the best effective treatments on the studied parameters and recorded the highest values in yellow color, while unfavorable treatments were recorded as follows: T6 < T1 < T3, as illustrated in Figure 5.

### 3.4. Multivariate Analysis: Based on Semithin and Ultrastructure Combined Data

#### 3.4.1. Correlation Matrix with Heat Map

**Figure 6:**
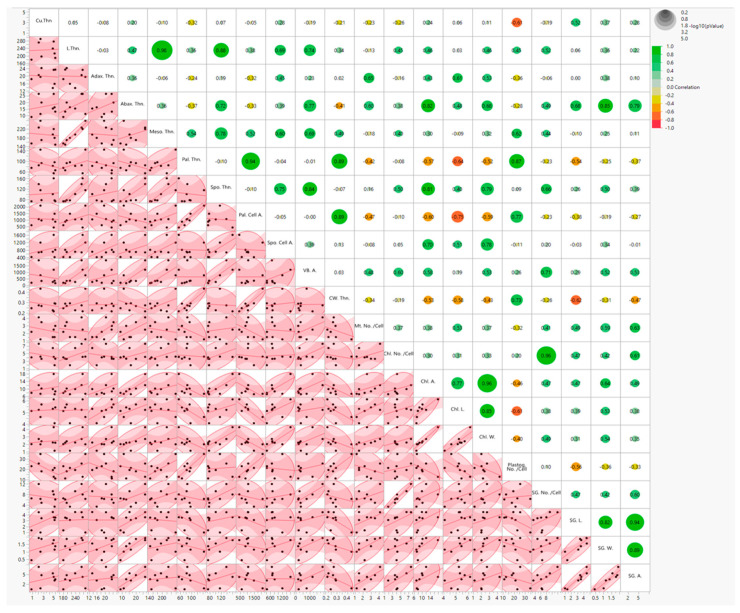
Scatter plot and heat map correlation matrix with outlier by turning circle scatter plot of 21 different semithin and ultra-structure parameters of 70-day-old *S. lycopersicum* plants under eight different treatments. Correlation levels were colored green for high levels of correlation and red for low levels of correlation (see scale in the upper right corner). The abbreviations were previously mentioned in the earlier figures.

Scatter plot with heat map correlation matrix showed that L. Thn. was weakly positively correlated with all the ultrastructural parameters (CW. Thn., Chl. No./Cell, Chl. A., Chl. L., Chl. W., Plastog. No./Cell, SG. No./Cell, SG. L., SG. W., and SG. A.) except Mt. No./Cell. In contrast, Adax. Thn. and Abax. Thn. were negatively correlated with Plastog. No./Cell, whereas Abax. Thn. were positively correlated with starch grain parameters, such as SG. No./Cell, SG. L., SG. W., and SG. A. as 0.49, 0.68, 0.85, and 0.79, respectively. Meso. Thn. had a positive linkage with ultrastructural parameters except Mt. No./Cell, Chl. L., and SG. L., while Pal. Thn. and Pal. Cell. A. showed a negative correlation with ultrastructural parameters except CW. Thn. (0.89) and Plastog. No./Cell as 0.87 and 0.77, respectively. Similarly, Spo. Thn. had a positive association with ultrastructural parameters except CW. Thn. and Spo. Cell. A. exhibited a strong positive correlation (0.70 and 0.78) with Chl. A. and Chl. W. Finally, VB. A. revealed a positive correlation with ultrastructural parameters, as represented in Figure 6.

#### 3.4.2. Principal Component Analysis (PCA)

**Figure 7:**
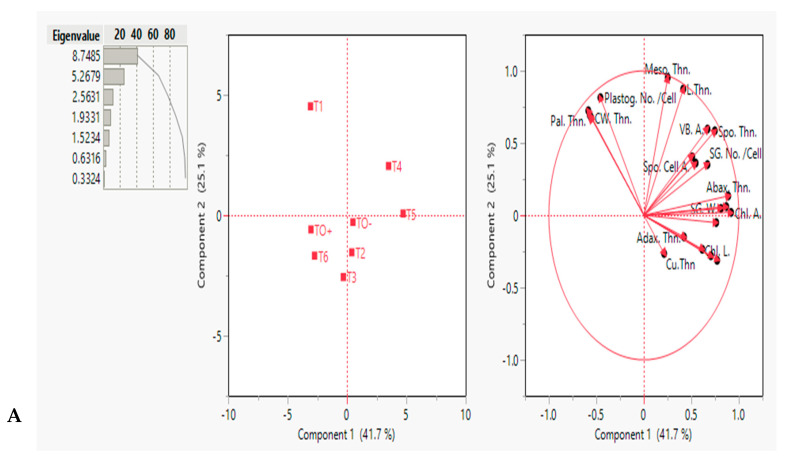

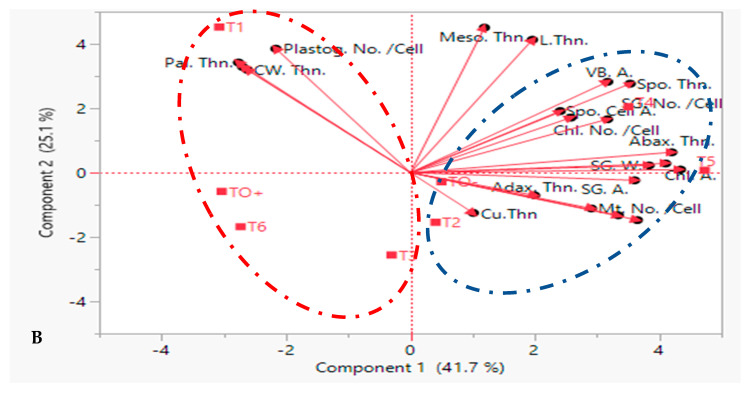
Principal component analysis (PCA-biplot). (**A**) Eigenvalues and scatter plots and (**B**) biplot illustrating the distribution of different treated *S. lycopersicum* plants on PC1 and PC2 components based on the analysis of semithin and ultrastructure traits. Blue and red dashed rings showed two different clusters. The dots were treatments, and the vectors (red arrows) were parameters. The abbreviations were previously mentioned in the earlier figures.

The PCA-biplot depicted interrelationships among the measured semithin and ultrastructural traits under various treatments, categorizing the eight treated *S. lycopersicum* plants into distinct clusters (Figure 7A). The first two principal components (PCs) accounted for 41.7% (PC1) and 25.1% (PC2) of the total variation. The treated plants were divided into two clusters: Cluster I (blue group) comprised T0−, T_2_, T_4_, and T_5_, whereas Cluster II (red group) included T0+, T_1_, T_3_, and T_6_. Objects with similar ordinates in the PCA scatterplot are more closely related, whereas those with dissimilar ordinates exhibit greater variation. The PCA-biplot (Figure 7B) showed that the vector lengths and cosines of the angles between them effectively discriminated against treatments. Pal. Thn., CW. Thn., and Plastog. No./Cell formed the longest vectors at a small angle, indicating a strong positive correlation. These traits were the most effective in differentiating the plants in Cluster II.

Conversely, vectors for Adax. Thn., SG.A., Mt. No./Cell, and Cu. Thn. distinguished between T0 and T_2_ plants in Cluster I. Abax. Thn., Sg. No./Cell, Spo. Thn., and VB. A. demonstrated strong discriminatory power for separating T_4_ and T_5_ plants in Cluster I. Vector correlations revealed the following additional insights: VB. A. and Spo. Thn. exhibited a strong positive correlation with Cu. Thn., which showed a negative correlation with CW. Thn. and Pal. Thn. Adax. Thn. displayed no correlation with the Mt. No./Cell, Meso. Thn., and L. Thn. The PCA-biplot clearly visualized how treatments influenced *S. lycopersicum* morphological traits, emphasizing the most effective parameters for distinguishing clusters.

## 4. Discussion

Plants frequently undergo significant physiological and anatomical changes when they encounter biotic stresses such as fungal infections. These alterations are not merely a consequence of pathogen-induced damage but also reflect the plant’s defensive mechanisms to mitigate stress effects. This study investigated the structural and ultrastructural modifications in *S. lycopersicum* leaves resulting from *F. oxysporum* infection and evaluated the efficacy of abiotic inducers (chemicals), thereby providing valuable insights into plant-pathogen interactions and stress responses.

This study examined the mesophyll anatomy of *S. lycopersicum* leaves infected with *F. oxysporum* using light microscopy. Infection (T0+) induced significant structural changes, underscoring its effect on leaf tissue expansion. For example, the reduction in phenolic content likely results from the increased volume of intercellular spaces due to the loosening of parenchyma cells [24]. This phenomenon led to a marked decrease in leaf blade thickness, mesophyll tissue thickness, and spongy intercellular space, contributing to the thinning of the leaf blade. Conversely, infection prompted a compensatory increase in cuticle thickness, as well as thickening of both the adaxial and abaxial epidermis, in comparison to non-infected control leaves (T0−). These structural modifications suggest a defensive response of tomato plants to fungal stress, with certain traits evolving to mitigate cellular damage, preserve structural integrity, and highlight the dynamic remodeling of leaf tissues under the influence of treatment.

At the ultrastructural level, pathogen infections frequently lead to distinct alterations in chloroplast shape, including enlarged chloroplasts, augmented starch accumulation, disintegrating grana stacks, stimulated stromule formation, and heightened plastoglobule accumulation [25]. Plastoglobuli (PGs) are plastid lipoprotein particles encased in a membrane lipid monolayer. PGs have small, specialized proteomes and metabolomes. PGs are involved in a variety of metabolic processes in response to environmental challenges such as excessive light, nitrogen shortage, and heat stress. However, their roles in biotic stress are mainly unknown. PGs are therefore lipid microcompartments that play various roles in plastid metabolism, developmental transitions, and environmental adaptability [26]. The PG metabolome and proteome are linked to four primary physiological modules [27]: chlorophyll breakdown, thylakoid remodeling, prenylquinone production, and carotenoid metabolism.

The number and distribution of PGs frequently fluctuate in response to biotic stressors such as infections [28]. For example, in plants inoculated with the necrotroph Botrytis cinerea, PG levels increased more than 20-fold, indicating that PGs play an active role in the plant’s response to biotic stress [29]. In this study, the maximum PGs were found in leaves treated with T1. These findings are explained by the fact that the most abundant proteins among the 30 core PG proteins are members of the plastid-specific fibrillin (FBN) family, which is anticipated to preserve the PG structure [30]. Studies have also linked FBN to disease resistance. The FBN1b ortholog in rice has also been related to stress resistance because of its interaction with SA glucosyltransferase (OsSGT1), an enzyme implicated in SA signaling [31]. *F. oxysporum* infection induced significant alterations in the chloroplasts, which could result in substantial changes in photosynthetic efficiency over prolonged infection periods. Such modifications may involve variations in phenolic content, potentially arising from direct effects on the synthesis or mobilization of phenolic compounds, or indirectly from changes in ultrastructural organization, including an increased chloroplast count per cell [32].

In *F. oxysporum*-positive control-treated plants, substantial ultrastructural changes were observed in chloroplast regions. Notable alterations included the detachment of chloroplast membranes from the cell wall, the disappearance of grana and starch grains, and a reduction in chloroplast size, which assumed a rounded shape. These structural degradations are accompanied by an increased number of plastoglobuli and a reduced number of starch granules. Such modifications are closely associated with a decline in photosynthetic attributes, likely because of reduced carbon fixation efficiency [33,34]. Comparable findings have been documented in rice subjected to salt stress, where chloroplasts exhibit enlarged plastoglobuli and reduced starch granules [35]. Similar patterns have been observed in maize exposed to elevated NaCl concentrations, resulting in an increase in plastoglobuli size and a decrease in both the size and number of starch granules [36]. Chernyad’ev [37] further emphasized that the proliferation of plastoglobuli and reduction of starch granules are indicative of chloroplast degradation, a characteristic of stress-induced damage to photosynthetic organelles. These observations are consistent with the findings of the current study and underscore the critical role of chloroplast ultrastructure in determining plant response to biotic stress.

While the thickening of epidermal cell walls offers a degree of protection, the infection caused significant structural alterations in the photosynthetic apparatus. Electron microscopy identified distinct microstructural characteristics in the mesophyll cells of the control leaves (T0−). The chloroplasts exhibited an ellipsoid shape accompanied by normal, small-sized mitochondria, a central vacuole, and an intact cellular membrane. The cell wall displayed a normal thickness, indicating a stable ultrastructure in the absence of infection. The breakdown of leaf chlorophyll, a critical catabolic process, is a reliable indicator of plant stress [38].

Mitochondria and vesicles are central to these defense mechanisms as they are involved in reactive oxygen species (ROS) production and transport, which are critical for stress signaling [39]. The increased number of mitochondria in *F. oxysporum*-infected plants is likely associated with the cellular demand for enhanced energy production, which supports stress responses and detoxification processes, as observed in Cd detoxification [40]. In this study, plasmolysis of the cell membrane, disturbances in the cell wall, and increased wall thickness were observed in the infected plants. Additionally, alterations in the nucleus, along with an increase in mitochondrial size and count, highlight cellular rearrangements associated with plant defense. These findings are in agreement with those of Pompeu et al. [41], who reported anatomical and ultrastructural changes in tomato leaves and roots subjected to Cd stress, including reduced intercellular spaces in shoots and altered organelle shape and membrane organization. Notably, the number of mitochondria and vacuoles increased in stressed roots, supporting the role of these organelles in energy production and storage under stress conditions.

The external application of salicylic acid (SA) triggers systemic acquired resistance (SAR) in plants against various pathogens. This response is associated with oxidative bursts, reinforcement of the cell wall, and changes in gene expression—either upregulation or downregulation [42]. In addition to slowing disease progression, SA also promotes the accumulation of phenolic compounds, which are known to contribute to plant resistance [43]. Recent studies have demonstrated that applying salicylic acid (SA) externally before inoculation enhances resistance to *Fusarium oxysporum* in Arabidopsis, as indicated by reduced leaf necrosis and lower plant mortality [44].

Additionally, Mandal et al. [45] reported that elevated levels of salicylic acid (SA) in root tissues following foliar application prior to *Fusarium oxysporum* f. sp. *lycopersici* (Fol) inoculation may have contributed to enhanced resistance, as evidenced by a significant reduction in vascular browning and leaf yellowing. Their findings suggest that SA can serve as an effective inducer of systemic acquired resistance (SAR) against the destructive soil-borne vascular wilt pathogen in tomato, primarily through activation of chemical defense mechanisms. This supports the hypothesis that SA initiates a signal transduction cascade leading to SAR expression, rather than directly suppressing fungal growth [46].

Moreover, humic acid has been shown to inhibit the growth and spore germination of several plant pathogenic fungi. This antifungal effect is attributed to its toxic constituents and functional properties, particularly its carboxyl group content and elemental makeup. Loffredo et al. [47] also reported that humic substances significantly reduced the radial growth and spore germination of *Fusarium oxysporum* f. sp. *melonis* and *Fusarium oxysporum* f. sp. *lycopersici*. In addition, De Azevedo et al. [48] proposed that humic acid (HA), when applied at low concentrations, can act as a plant biostimulant by enhancing growth, nutrient uptake, and overall yield. Similarly, El-Fawy et al. [49] investigated the effects of humic acid, L-methionine, and phosphoric acid on managing Fusarium wilt and activating oxidative stress-related defense enzymes in tomato plants. Among the treatments, T4 exhibited the largest vascular bundle area, which may be attributed to HA’s ability to help plants retain more water in their cells, thereby mitigating the negative impacts of biotic stress. These findings are consistent with the results reported by Khan et al. [50].

In this study, notable enhancements were observed in the chloroplast and mitochondrial structures of T4-treated leaves. These results are consistent with previous research showing that humic acid (HA) promotes leaf expansion and greenness, increases chlorophyll content, and enhances light absorption and photosynthetic efficiency in plants under stress [51]. Additionally, T3 treatment led to increased cell wall thickness and improved membrane stability. These findings align with those of Khan et al. [50], who reported enhanced membrane integrity in tomato plants treated with *Fusarium solani* IK-105 and HA, suggesting a potential role in mitigating lead (Pb) toxicity. Furthermore, both T3 and T4 treatments showed the presence of microsomes of varying sizes, which may be linked to HA-induced activation of catalase and peroxidase enzymes, as supported by the findings of Amoozad and Zahedi [52].

In the present study, the largest chloroplast area was observed in tomato leaves treated with T5. Previous research supports these findings, as nano-ZnO treatments have been shown to increase chlorophyll and protein content in *Triticum aestivum* [53] and to elevate levels of glycyrrhizin, total phenolics, and anthocyanins in licorice (*Glycyrrhiza glabra* L.) seedlings compared to their bulk counterparts [54]. Also, Abdelaziz et al. [55] investigated the protective effects of a ZnO nanoparticle-based hydrogel against wilt disease in pepper plants caused by *Fusarium oxysporum*. Similarly, Imran et al. [56] found that ZnO-NPs significantly reduced the severity of gray mold disease in tomatoes while enhancing catalase and peroxidase enzyme activity. In a more recent study, Bouqellah et al. [57] demonstrated that ZnO-NPs not only protected tomato plants from Fusarium wilt but also promoted the production of biochemical compounds involved in plant defense. Furthermore, Jomeyazdian et al. [58] reported that foliar application of synthesized ZnO-NPs effectively increased zinc concentrations in tomato leaves, helping to alleviate zinc deficiency. Their findings also showed a significant reduction in disease severity under in vivo conditions, with green-synthesized ZnO-NPs outperforming conventional fungicides at lower concentrations.

These structural abnormalities are consistent with the findings of the present study in ZnO NP-treated plants, where circular mitochondria were prominent and the counts of chloroplasts and starch granules improved, thereby mitigating the effects of *F. oxysporum* infection. Similarly, treatment with SA (0.6 mM), HA (150 mg/L), and ZnO NPs (250 mg/L) exhibited protective effects, as indicated by ultrastructural features suggesting reduced fungal damage. These results corroborate previous findings regarding the efficacy of SA and ZnO NP treatments in enhancing the structural resilience of rapamycin and eggplant against pathogen-induced stress [59,60]. These findings were supported by multivariate analyses of integrating data from 21 semi-thin and ultrastructural parameters collected at 70 days. These analyses employed principal component analysis (PCA), utilizing both scatter and biplots for comprehensive visualization of traits. The analysis identified distinct groupings of treated plants into two clusters, highlighting the shared patterns and unique differentiation across treatments. These clusters were well-supported by the analyses, underscoring the efficacy of multivariate approaches in distinguishing traits associated with varying treatment effects. These treatments underscore promising avenues for developing sustainable chemical inducer strategies to manage *F. oxysporum* infection.

## 5. Conclusions

The results of this study underscore the potential of salicylic acid, humic acid, and ZnO nanoparticle treatments as effective chemical inducers strategies to combat *F. oxysporum*. These treatments not only alleviate infection-induced damage but also enhance plant growth and functional capacity. Practical applications of these inducers can be integrated into sustainable agricultural practices to promote disease resistance in tomato crops and reduce their dependence on chemical fungicides. Future studies should focus on optimizing the treatment concentrations to maximize efficacy and minimize potential adverse effects. Investigating the underlying molecular mechanisms governing observed anatomical and ultrastructural changes will provide deeper insights into plant stress responses and pathogen resistance. Moreover, expanding the research to assess chemical inducer performance across diverse environmental conditions and crop varieties will ensure broader applicability and effectiveness. Exploring the synergistic use of multiple treatments could further enhance plant resilience to fungal pathogens.

## Figures and Tables

**Table 1:** Morphometric assessments of leaf semithin section measurements of treated and untreated *S. lycopersicum*.

Treatment/Leaf Parameters	Leaf Blade Thickness (µm)	Cuticle Thickness (µm)	Adaxial Epidermis Thickness (µm)	Abaxial Epidermis Thickness (µm)	Mesophyll Thickness (µm)	Palisade Thickness (µm)	Spongy Thickness (µm)	Palisade Parenchyma Area (µm^2^)	Spongy Parenchyma Area (µm^2^)	Vascular Bundle Area (µm^2^)
T0−	239.02 ± 8.28 bc	4.62 ± 0.13 c	13.63 ± 0.31 a	15.37 ± 0.36 c	195.31 ± 5.64 cd	82.18 ± 1.90 c	100.46 ± 3.48 b	1049.79 ± 30.31 d	711.29 ± 20.53 bc	256.94 ± 8.90 abc
T0+	212.71 ± 7.37 ab	4.94 ± 0.14 c	15.86 ± 0.37 a	16.31 ± 0.38 c	179.81 ± 5.19 bc	96.16 ± 2.22 e	76.51 ± 2.65 a	1479.93 ± 42.72 e	422.08 ± 12.18 a	351.18 ± 12.16 c
T1	290.35 ± 10.06 d	2.40 ± 0.07 b	19.59 ± 0.45 b	11.21 ± 0.26 b	258.40 ± 7.46 f	144.39 ± 3.33 f	115.49 ± 4.00 b	2102.60 ± 60.70 f	1203.83 ± 34.75 d	768.79 ± 26.63 d
T2	196.37 ± 6.80 a	2.58 ± 0.08 b	24.32 ± 0.56 c	19.97 ± 0.46 d	169.98 ± 4.91 abc	92.83 ± 2.14 de	77.47 ± 2.68 a	967.65 ± 27.93 cd	697.17 ± 20.13 bc	335.47 ± 11.62 bc
T3	188.93 ± 6.54 a	5.06 ± 0.14 c	23.48 ± 0.54 c	12.99 ± 0.30 b	148.84 ± 4.30 a	65.35 ± 1.51 b	73.28 ± 2.54 a	872.40 ± 25.18 c	815.76 ± 23.55 c	164.28 ± 5.69 a
T4	271.32 ± 9.40 cd	2.09 ± 0.06 b	20.53 ± 0.47 b	26.59 ± 0.61 e	227.42 ± 6.57 e	84.56 ± 1.95 cd	138.71 ± 4.80 c	1068.66 ± 30.85 d	677.14 ± 19.55 b	1913.68 ± 66.29 f
T5	276.58 ± 9.58 cd	4.78 ± 0.14 c	23.37 ± 0.54 c	26.56 ± 0.61 e	220.48 ± 6.37 de	54.02 ± 1.25 a	160.85 ± 5.57 d	427.98 ± 12.36 a	1614.09 ± 46.60 e	1192.74 ± 41.32 e
T6	179.42 ± 6.22 a	0.78 ± 0.02 a	18.40 ± 0.42 b	8.90 ± 0.20 a	154.38 ± 4.46 ab	74.62 ± 1.72 bc	72.97 ± 2.53 a	602.64 ± 17.40 b	428.85 ± 12.38 a	194.97 ± 6.75 ab

Values are represented as the mean of five replications. Means followed by different letters are significantly different at *p* ≤ 0.05 (Post Tukey’s (HSD) test). Treatments: T0−: Negative control (dist. water); T0+: Positive control (*Fusarium oxysporum*); T1: SA (0.5 mM) + *F. oxysporum*; T2: SA (0.6 mM) + *F. oxysporum*; T3: HA (100 mg/L) + *F. oxysporum*; T4: HA (150 mg/L) + *F. oxysporum*; T5: ZnO-NPs (250 mg/L) + *F. oxysporum* and T6: ZnO-NPs (500 mg/L) + *F. oxysporum*.

**Table 2:** Quantitative parameters of ultrastructure of treated and untreated *S. lycopersicum* leaves.

Quantitative Parameters/Treatments	Cell Wall Thickness (µm)	Mitochondria Number/Cell Profile	Chloroplast Number/Cell Profile	Chloroplast Area (µm^2^)	Chloroplast Length (µm)	Chloroplast Width (µm)	Plastoglobuli Number/Cell Profile	Starch Grain Number/Cell Profile	Starch Grain Length (µm)	Starch Grains Area (µm^2^)
T0−	0.23 ± 0.01 a	1.50 ± 0.08 a	6.50 ± 0.33 c	9.89 ± 0.49 b	5.31 ± 0.27 bc	2.25 ± 0.11 bc	19.38 ± 0.97 bc	9.25 ± 0.46 e	4.50 ± 0.23 d	5.35 ± 0.27 d
T0+	0.29 ± 0.02 c	1.50 ± 0.09 a	1.90 ± 0.11 a	7.13 ± 0.43 a	4.08 ± 0.25 a	1.15 ± 0.07 a	21.33 ± 1.28 cd	3.33 ± 0.20 a	3.13 ± 0.19 b	3.15 ± 0.19 b
T1	0.43 ± 0.02 d	1.20 ± 0.05 a	4.20 ± 0.17 b	7.35 ± 0.29 a	4.61 ± 0.18 ab	2.01 ± 0.08 b	34.64 ± 1.39 f	6.30 ± 0.25 c	0.99 ± 0.04 a	0.75 ± 0.03 a
T2	0.27 ± 0.01 bc	3.50 ± 0.14 d	3.60 ± 0.14 b	10.18 ± 0.41 b	5.69 ± 0.23 cd	2.41 ± 0.10 cd	18.10 ± 0.72 b	4.80 ± 0.19 b	3.44 ± 0.14 bc	4.79 ± 0.19 cd
T3	0.29 ± 0.01 c	3.00 ± 0.15 c	3.64 ± 0.19 b	8.65 ± 0.43 ab	5.43 ± 0.28 cd	2.22 ± 0.11 bc	11.27 ± 0.57 a	6.10 ± 0.31 c	3.55 ± 0.18 bc	3.53 ± 0.18 b
T4 large SG	0.27 ± 0.01 bc	4.14 ± 0.17 e	7.64 ± 0.31 d	11.99 ± 0.48 c	5.23 ± 0.21 bc	2.72 ± 0.11 d	25.55 ± 1.03 e	12.00 ± 0.48 f	4.47 ± 0.18 d	7.31 ± 0.29 e
T4 small SG	--	---	--	--	--	--	--	--	1.36± 0.08 b	0.85 ± 0.02 c
T5	0.22 ± 0.02 a	2.50 ± 0.15 b	4.13 ± 0.25 b	17.96 ± 1.08 d	6.13 ± 0.37 d	4.30 ± 0.26 e	12.86 ± 0.77 a	8.00 ± 0.48 d	3.61 ± 0.22 c	4.25 ± 0.26 c
T6	0.25 ± 0.02 ab	2.20 ± 0.11 b	4.00 ± 0.20 b	7.85 ± 0.39 a	5.13 ± 0.26 bc	2.03 ± 0.10 b	23.00 ± 1.15 de	6.50 ± 0.33 c	1.35 ± 0.07 a	0.46 ± 0.03 a

One-way ANOVA test was performed using (Post Tukey’s (HSD) test), the results were recorded as mean of triplicates ± standard Deviation (SE). Different letters refer to significant differences at (*p* ≤ 0.05).

## Data Availability

All data generated or analyzed during this study are included in this published article.
